# Local and Landscape Drivers of Carabid Activity, Species Richness, and Traits in Urban Gardens in Coastal California

**DOI:** 10.3390/insects10040112

**Published:** 2019-04-19

**Authors:** Stacy M. Philpott, Simone Albuquerque, Peter Bichier, Hamutahl Cohen, Monika H. Egerer, Claire Kirk, Kipling W. Will

**Affiliations:** 1 Environmental Studies Department, University of California, Santa Cruz, CA 95062, USA; pbichier@ucsc.edu (P.B.); megerer@ucsc.edu (M.H.E.); clairekirk95@gmail.com (C.K.); 2 Ecology and Evolutionary Biology Department, University of California, Santa Cruz, CA 95062, USA; lostinalbuquerque@gmail.com; 3 Entomology Department, University of California, Riverside, CA 92521, USA; hamutahc@ucr.edu; 4 Essig Museum of Entomology, University of California, Berkeley, CA 94720, USA; kipwill@berkeley.edu

**Keywords:** Carabidae, California, local vs. landscape, ground beetle, urban gardens

## Abstract

Urban ecosystems, as mosaics of residential, industrial, commercial, and agricultural land, present challenges for species survival due to impervious surface, degradation, fragmentation, and modification of natural habitat, pollution, and introduced species. Some urban habitats, such as community gardens, support biodiversity and promote ecosystem services. In gardens, local factors (e.g., vegetation, groundcover) and landscape surroundings (e.g., agriculture, built or impervious cover) may influence species abundance, richness, and functional traits that are present. We examined which local and landscape factors within 19 community gardens in the California central coast influence ground beetle (Carabidae) activity density, species richness, functional group richness, and functional traits—body size, wing morphology, and dispersal ability. Gardens with higher crop richness and that are surrounded by agricultural land had greater carabid activity density, while species and functional group richness did not respond to any local or landscape factor. Gardens with more leaf litter had lower carabid activity, and gardens with more leaf litter tended to have more larger carabids. Changes in local (floral abundance, ground cover) and landscape (urban land cover) factors also influenced the distribution of individuals with certain wing morphology and body size traits. Thus, both local and landscape factors influence the taxonomic and functional traits of carabid communities, with potential implications for pest control services that are provided by carabids.

## 1. Introduction

Impervious land cover, habitat degradation and modification, and fragmentation spur biodiversity loss within urban areas [1,2]. Yet, depending on local or landscape characteristics, urban habitats may support taxonomically and functionally rich communities of arthropods [3,4] and associated ecosystem services. The relative importance of local and landscape drivers of urban biodiversity varies for different organisms, such as arthropods [5]. At the local habitat scale, arthropod abundance and species richness increase with plant richness and woody plant presence [6,7]. At the landscape scale, natural vegetation cover enhances arthropod abundance and species richness [8,9,10]. In contrast, impervious surface (i.e., concrete) negatively affects arthropods, including pollinators and natural enemies [11,12,13,14]. Species life history and functional traits—phenotypes that affect ecosystem processes [15]—can also determine how local and landscape scale changes in urban environments drive community formation [3,4]. Feeding habits, habitat preference, body size, and dispersal ability are traits that may be vary in sensitivity to local and landscape factors. For example, changes in leaf litter differentially affect cavity- and ground-nesting bees [16]. Increases in impervious cover more strongly affect light-preferring than xerophilous spiders [17], and negatively impact spiders with high dispersal ability [14]. Thus, landscape-scale urbanization and local habitat management can selectively filter for certain traits, thereby structuring urban communities [3,18]. Changes in both taxonomic and functional richness and the traits of individuals within communities are important to monitor because arthropods provide ecosystem services. Thus, understanding to what extent local and landscape factors affect arthropods informs both conservation and function [19]. 

Beetles (Order: Coleoptera) are abundant, diverse, and play important roles in urban ecosystems [13,20,21,22], but carabid diversity and community composition vary along urban to rural gradients and carabid functional traits (e.g., body size, wing morphology) vary with local and landscape factors [23]. Beetles are natural enemies, detritivores [24], and bioindicators of ecosystem-level processes [21,25,26]. In particular, ground beetles (Carabidae) are sensitive to environmental changes, taxonomically and functionally diverse, easy to sample, and are often used in ecological research [25,27,28]. Carabids respond to changes in landscape forest cover [28,29] and to local agroecosystem management, such as hedgerow or field margin planting [30,31,32]. As carabids might positively respond to intermediate levels of urbanization, urban ecosystems may conserve relatively high species diversity when compared to more natural habitats [33]. 

Carabid traits (wing morphology and body size) and landscape connectivity and quality influence the dispersal and distribution of carabids [34], influencing habitat colonization across urbanization gradients [23]. Individual carabid species have three types of wing development and dispersal ability: (1) monomorphic brachypterous (reduced wings; low dispersal ability); (2) monomorphic macropterous (fully developed wings; high dispersal ability); and, (3) dimorphic or polymorphic (a range of wing types; variable dispersal ability) [35,36]. High dispersal species are common in farms, prairies, and highly disturbed habitats, and low dispersal species are associated with older, less disturbed habitats [37,38]. Smaller carabids may disperse farther, depending on wing morphology, and they are more abundant in areas with highly degraded, modified, and fragmented habitats [33,39,40,41,42]. Yet, in agroecosystems, larger carabid species consume more prey and provide better pest control [43]. Thus, environments with fewer large carabids may experience less pest control. An impervious surface may be an environmental filter of carabid functional traits, like body size [42,44,45], but less is known regarding how local management and landscape surroundings affect carabid activity, species richness, and functional traits in urban ecosystems.

Urban agroecosystems provide an ideal system to examine the drivers of carabid taxonomic and functional diversity, community composition, and traits. Gardens support biodiversity [46,47] and bridge habitat conservation with food production and community development [48]. Differences in urban habitat composition and structure influence carabid activity [49], diversity [37], and may result in changes in the abundance of beetles with certain traits [50]. Although urbanization generally leads to biodiversity loss, it is important to determine what urban habitats, and which characteristics of those habitats, can support biodiversity conservation in the future. To this end, we compared activity density, species richness, functional group richness, and traits (body size, wing morphology, dispersal ability) of carabids in urban community gardens that differ in local (e.g., vegetation and ground cover) and landscape (percent cover from different land use types, including impervious cover) features. In order to determine how gardeners might promote carabid activity and taxonomic and functional richness for conservation purposes or to promote ecosystem services that are provided by carabids, we examined: (1) Which local habitat and landscape features of urban, community gardens influence carabid activity density, species richness, and functional group richness? and, (2) Which local habitat and landscape features of urban, community gardens influence carabid community and trait composition?

## 2. Materials and Methods

### 2.1. Study Sites

We sampled carabids during a single growing season between May–September 2013 in 19 urban, community gardens in Monterey, Santa Clara, and Santa Cruz Counties in the California central coast. At the time of the research, the gardens ranged in size from 444 m² to 15,525 m², were between 4 to 46 years old, and they were separated from one another by >2 km. We chose gardens that varied in local factors (e.g., vegetation composition and structure, ground cover) and landscape surroundings (e.g., land area in agriculture, urban developed land, and natural habitat).

### 2.2. Local and Landscape Factors

We measured 38 local factors monthly within 20 × 20 m plots in the center of each garden. We sampled the canopy cover with a concave vertical densiometer at the center and 10 m to the N, S, E, and W of the center. We counted and identified trees and shrubs >2 m tall, and noted the number of trees and shrubs in flower. Within 20 × 20 m plots, we randomly placed four 1 × 1 m plots within which we measured the height of the tallest vegetation, assessed crop, ornamental, and weed richness, counted flowers, and visually estimated the percent cover of (a) bare ground, (b) grass, (c) herbaceous plants, (d) rocks/wood panels, (e) leaf litter, (f) mulch, and (g) concrete.

We used the United States (US) Geological Survey 2011 National Land Cover Database (NLCD, 30 m resolution) [51] data to measure land cover types within 2 km of each study site. We used land cover type data to create four landscape variables: (1) natural habitat area (including deciduous evergreen, and mixed forests, dwarf scrub, shrub/scrub, and grassland/herbaceous), (2) open area (including lawn grass, parks, and golf courses), (3) urban area (including low, medium, and high intensity developed land), and (4) agriculture area (including pasture/hay and cultivated crops). We excluded land cover types (open water, wetlands, and barren land) that covered <5% of the area in buffer zones. Of all 19 sites sampled, ten had no agriculture within 2 km, seven had between 1–10% agriculture in the landscape, and just two had >10% agriculture in the landscape. Most agriculture in the landscapes surrounding the study sites are intensive monoculture strawberry fields that are managed with conventional practices. 

### 2.3. Carabid Sampling, Identification, and Traits

We sampled carabids with pitfall traps for 72 h in each site monthly (20–23 May, 17–20 June, 15–18 July, 11–14 August, and 9–12 September). The pitfall traps indicate carabid beetle activity density, not necessarily abundance [23]. Pitfall traps were made of 12 oz. clear plastic tubs (11.4 cm diameter × 7.6 cm deep). We placed traps at the center of the 20 × 20 m plots in two rows of three traps, and separated each trap by 5 m. We buried traps flush to the soil level and filled traps with 200 mL of a saturated saline solution with a drop of unscented detergent to break the surface tension. We placed green plastic plates (7.62 cm diameter) over each trap and elevated plates 7–8 cm above the ground with nails to limit the rainwater influx and to capture non-target taxa. Upon collection, we rinsed arthropods with water, separated them to order, and then stored insects in vials with 70% ethanol. Our sampling effort was unfortunately not as high as some other studies that examined carabids along urbanization gradients [39,42,52,53]. We placed pitfall traps in active garden beds (where gardeners otherwise were tending crop and ornamental plants). Thus, we were unable to leave traps out for longer than 72 h or to get permission for putting traps more than three times during the summer growing season. 

KWW at the Essig Entomology Museum at the University of California, Berkeley (EMEC), used published keys and descriptions [36] and a comparison to authoritatively identified specimens in EMEC to identify the beetles. Nomenclature follows Lorenz (2018). For each individual, we measured body length (mm) and grouped beetles into small (<8 mm) and large (>8 mm) size classes [25]. We determined the flight wing morphology for each beetle by lifting the elytra under a dissecting microscope and noting wing state. We classified beetles as macropterous if the wing length was equal to abdomen length and the wings were folded at the apex or longer and as brachypterous if the wings were reduced or not apparent. We did not examine flight muscles. Thus some beetles categorized as macropterous based on wing length may not be able to fly. Carabid species were classified as monomorphic if all of the individuals had the same wing type and dimorphic if individuals had both wing types. We used body length and wing morphology as a surrogate for dispersal ability and designated three groups: (1) large beetles with brachypterous wings as low dispersal ability; (2) large beetles with macropterous wings, or small beetles with brachypterous wings as medium dispersal ability; and, (3) small beetles with macropterous wings as high dispersal ability.

We used three functional traits—size (small, large), wing morphology (macropterous, brachypterous), and wing syndrome (monomorphic, dimorphic)—to assign beetles to functional groups. The individual functional groups were based on unique combinations of trait values for a total of eight possible functional groups. We used the number of functional groups that are present in each garden as a measure of functional group richness.

### 2.4. Statistical Analysis

All of the statistical analysis was conducted in R version 1.1.456 [54]. To determine how activity density, species richness, and functional group richness vary with the local and landscape factors, we used generalized linear models (GLMs) and a model selection approach based on Akaike’s Information Criterion (AICc). We determined total activity density, species richness, average body size, dispersal class activity density, and functional group richness for each site across all of the sampling periods. Rather than include all local and landscape variables measured, we ran Pearson’s correlations to select variables that were uncorrelated and biologically relevant given other studies on carabids. Thus, we included garden size (natural log), county (Monterey, Santa Clara, Santa Cruz), percent bare soil cover, percent mulch cover, percent leaf litter cover, floral abundance (natural log), number of crop species, number of weed species, and the amount of urban land cover (square root) within 2 km, and amount of agriculture land cover (square root) within 2 km as the explanatory variables for each model. To determine the landscape scale to use in the model, we performed stepwise model selection comparing the model fits at each scale. We selected 2 km because it had the best model score and is a comparable scale to carabid studies in other systems [37,55]. Although garden age might impact carabid communities (e.g., [56]), we did not include garden age because this factor positively correlates with garden size. We did not include any random terms in the models. The models were fit with Poisson error distributions. We used the “glmulti” package version 1.0.7 [54,57] to identify the best fit model using AICc. If the best fitting models differed by <2 points, we averaged the top models (up to 10 models). Models with significant predictors of variables were visualized with the “visreg” package version 2.5-0 [58].

To assess which local and landscape factors drive carabid community composition, we examined the patterns and graphics with the “vegan” package version 2.5-3 [59]. We used a permutational multivariate analysis of variance (PERMANOVA) with the “adonis2” function. We calculated the Bray–Curtis distance and used the “metaMDS” function to transform and visualize the community structure in each garden. We included the county (Monterey, Santa Clara, Santa Cruz) as a random factor. To visualize the results, we plotted non-metric multidimensional scaling (NMDS) plots with the “ordiplot” function, and used the “envfit” function to fit the local and landscape factors to the ordination.

To determine how local and landscape factors influence carabid traits, we used a combined RLQ and a fourth corner approach with the “ade4” package version 1.7-11 [60]. We used the RLQ method to summarize the joint structure between the local and landscape factors, carabid distribution among gardens, and carabid traits, and then used the fourth corner to test for correlations between local and landscape factors and carabid traits [61,62]. We created three matrices: R matrix (local and landscape factors), Q matrix (carabid traits), and L matrix (species abundances). We performed a correspondence analysis (L matrix) and principal component analysis (R, Q matrices) and then used two permutation models to evaluate whether garden factors influence the distribution of carabid traits (model 2), and if traits influence the composition of species assemblages that are found in gardens (model 4) [61]. We created an RLQ biplot to assess the relationships between species traits and local and landscape factors and determined the significance of each trait-factor relationship using the fourth corner analysis. For trait analyses, we removed the singleton species and transformed species abundance with a Hollinger transformation [63]. We included the same local and landscape factors that were used in the GLMs for activity and taxonomic richness. We used Monte-Carlo permutations (9999) to test for correlations between quantitative variables and used the “D2” correlation coefficient to test for associations between quantitative variables and each categorical value separately [61]. 

## 3. Results

### 3.1. Local and Landscape Drivers of Carabid Activity and Richness

We collected 149 carabid individuals from 14 genera and 20 species (Table 1). *Trechus obstusus* was the most abundant (34.2% of individuals), followed by *Laemostenus complanatus* (Dejean) (21.5%), *Pterostichus californicus* (Dejean) (10%), and *Harpalus pensylvanicus* (DeGeer) (5.3%). We recorded low abundance (<5 % of individuals) of several species that often occupy disturbed habitats, including *Microlestes nigritus* (Mannerheim) and *Axinopalpus biplagiatus* (Dejean) [64,65]. We collected two species that feed on seeds and pollen—*Bradycellus nubifer* (LeConte) and *B. nitidus* (Dejean) [66,67]. Carabids varied in body length (from 2–21 mm). Two species exhibited dimorphic wing—*M. nigritus* (one brachypterous, two macropterous individuals) and *T. obtusus* (18 brachypterous, six macropterous individuals); all other species were monomorphic (Table 1). Carabids were low (two species, 19 individuals), medium (13 species, 113 individuals), or high (five species, 17 individuals) dispersers (Table 1).

**Figure 1:**
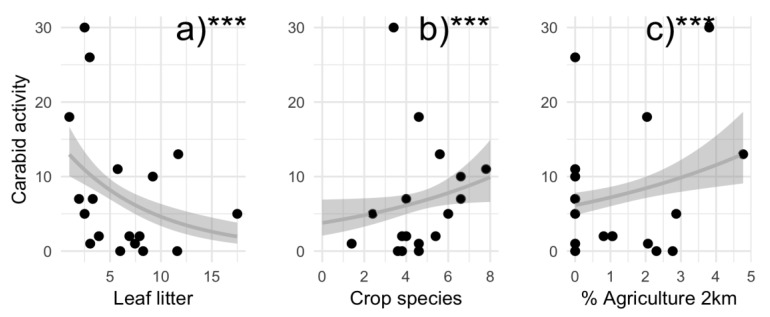
Local and landscape drivers of carabid activity density in urban gardens in the California central coast as determined with generalized linear models. Carabid activity responded to leaf litter (**a**), crop species richness (**b**), and agriculture land cover within 2 km surrounding gardens (**c**).

Local and landscape factors predicted the activity density, species richness, and functional group richness. Carabid activity density increased with crop richness (GLM, Z = 8.44, *p* < 0.001) and agriculture cover (GLM, Z = 7.43, *p* < 0.001), but declined with leaf litter (GLM, Z = −6.24, *p* < 0.001; Figure 1). The best model predicting carabid species richness included weed and crop species richness, but no factors significantly influenced carabid species richness. The model that best predicted carabid functional group richness included the number of weed and crop species, leaf litter, bare soil, and urban land cover, but no factor significantly predicted carabid functional group richness. 

### 3.2. Local and Landscape Drivers of Community and Trait Composition

**Figure 2:**
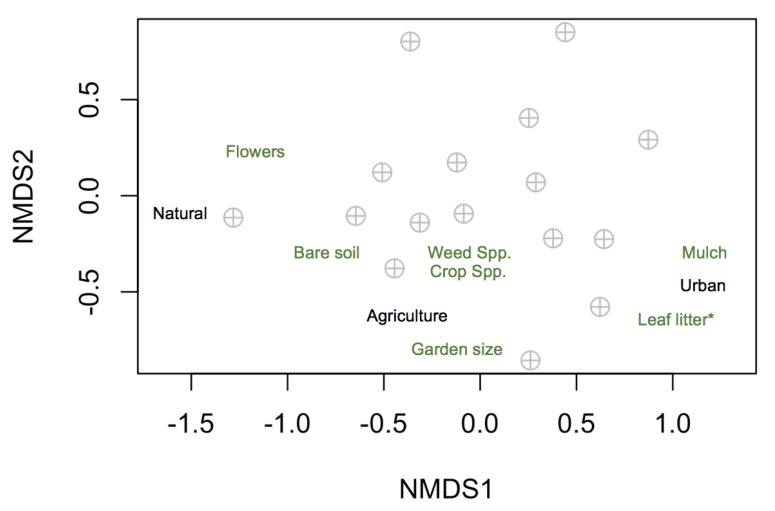
Non-metric multidimensional scaling plot showing carabid community composition in the sixteen gardens where beetles were collected in relation to local and landscape factors. The asterisk (*) indicates the one significant factor.

Leaf litter was the only significant driver of Carabidae community composition of species in the gardens (PERMANOVA, F_1,6_ = 2.308, *p* = 0.01; Figure 2).

**Figure 3:**
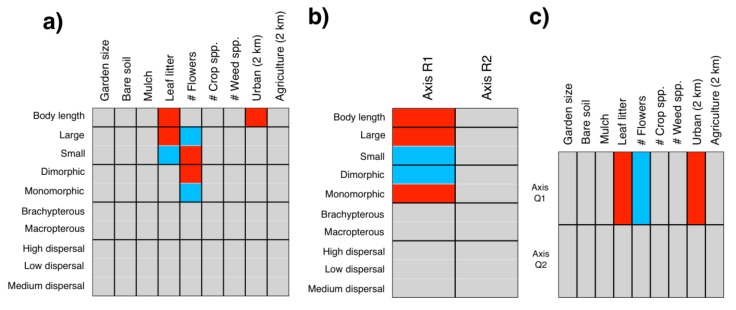
Biplot of fourth corner analysis (**a**) of carabid traits in relation to local and landscape factors. In (**b**) and (**c**), biplot of RLQ analysis of the relationship between the carabid trait axis (Q) in relation to the local and landscape factors axis (R). Red indicates positive and blue indicates negative correlations between factors.

Local and landscape factors influenced species trait distributions (model 2: *p* = 0.036; model 4: *p* = 0.037). Local factors were positively (number of flowers) and negatively (leaf litter) associated with the first trait axis (AxQ1), and this axis relates to body length (Table S1; Figure 3). One landscape-scale factor, urban land cover, was negatively associated with AxQ1. Body length was negatively associated with the first local and landscape factor axis (AxR1). The correlation matrix derived from the fourth corner analysis detected six significant correlations between local and landscape factors and carabid functional traits (Table S2; Figure 3). Carabid body length was higher with more leaf litter (*p* = 0.005) and more urban land cover (*p* = 0.03), but body length declined with floral abundance (*p* = 0.05). Small beetles were less abundant with more leaf litter (*p* = 0.04), but they were more abundant with more flowers (*p* = 0.04). Beetles with dimorphic wing morphology were more abundant with more flowers (*p* = 0.01). 

## 4. Discussion

The urban gardens that we studied support many carabid species, agreeing with previous findings that urban ecosystems can harbor high carabid richness (e.g., [68]). We found relatively low activity density of carabids (only 149 individuals trapped across a summer), which potentially indicates that high levels of fragmentation, urbanization, and habitat modification in the study region may affect carabid abundance at the regional scale, and that sampling may not have been extensive enough. We found two studies that examined carabid abundance in natural or semi-natural habitats in Coastal California, although the two studies differed in trap type and sample effort. One study that was conducted in coastal sage scrub and coastal chaparral fragments in Southern California used similar traps to ours and found between 0.7–9.3 carabid beetles per trap per day [69]. A second study conducted in perennial bunchgrass prairie site in Northern California used directional pitfall traps to trap beetles for three weeks and found 220 *Poecilus diplophryus* Chaudoir (under the synonym *Pterostichus subcordatus* (LeConte)) and 533 *Dicheirus dilatatus* (Dejean) individuals and between 1–79 individuals of other carabid and tenebrionid species [70]. They collected between 0.09–2.47 beetles (both carabids and tenebrionids) per trap per day. In our study, we collected 1.02 carabids per trap per day, and thus our results are comparable in activity density with these two other studies in relatively nearby natural habitats. Furthermore, two of our garden sites were small (444 m^2^ or 20 × 22 m; 654 m^2^ or 25 × 26 m), such that pitfall traps were placed < 10 m from the nearest habitat edges. All other gardens were at least 1600 m^2^, and the pitfalls were placed ≥ 20 m from a habitat edge. One factor that may influence carabid abundance and diversity is dispersal from nearby habitats [71,72]. Thus it is important to acknowledge that, in these small spaces, edge effects may have introduced potential bias into the results, for example, by filtering carabid community composition or by serving as impermeable barriers [73]. Even with relatively low numbers of beetles captured, and the potential for edge effects, garden vegetation, groundcover management, and landscape surroundings significantly influenced carabid activity. Moreover, many of the local and landscape factors that influenced activity correlated with changes in the activity of beetles with certain body size and wing morphology traits, with both being important in dispersal ability. The life histories of the species that we found likely explain the differences in relationships between local and landscape factors and biodiversity measures.

### 4.1. Local and Landscape Drivers of Carabid Activity and Richness

Carabid activity density responded to several local factors and one landscape factor. Local factors, including crop species richness and leaf litter influenced carabid activity, and they were important in models predicting species and functional group richness. A diverse crop assemblage could provide food and shelter (i.e., structural heterogeneity), promoting carabid activity in gardens. Crop species richness may benefit carabids by directly providing an array of seeds, fruit, and pollen [25,74,75]. In rural agriculture, seed additions increase the abundance of seed-feeding carabids [76]. In addition, crop diversity could indirectly attract and support carabid richness by providing habitat and resources for carabid prey [25,77,78]. Documented increases in carabid activity density with more leaf litter corroborate previous results (e.g., [79,80]). Interestingly, landscape factors predicted carabid activity but not species richness in gardens. Agricultural land cover positively correlated with carabid activity, potentially due to high activity of *T. obtusus*, a species frequently associated with agriculture [38,81,82]. 

We did not find significant local or landscape predictors of species or functional group richness, which was perhaps due to influences on species traits. Species traits were correlated with urban land cover, suggesting that carabids with different trait combinations persist in urban areas, and highlighting the importance of considering functional group richness and trait composition in arthropod communities.

### 4.2. Local and Landscape Drivers of Community and Trait Composition

Specific local and landscape features influenced carabid traits in the gardens. Ground cover features and flowers were important for carabids across multiple analyses. In our study, community composition significantly influenced leaf litter cover, and larger carabids associated with sites with more leaf litter. In forest systems, carabid composition can differ with natural variation or manipulation of litter depth (e.g., [83,84]). At least two studies have found larger carabid body size in forest sites with more litter [85,86]. However, not all sites document the differences in carabid communities or traits with changes in litter depth along urban to rural gradients (e.g., [87]). Leaf litter may influence carabids by providing additional prey resources, or it may strongly alter microhabitat conditions [83]. Larger beetles utilize leaf litter for shelter and gardens with more leaf litter may provide refuges from predation. In contrast, smaller beetles may have difficulty moving across areas with high leaf litter. Large carabids with reduced wings were associated with gardens with more leaf litter. Smaller carabids with high dispersal ability were associated with high floral abundance, as were carabid species with dimorphic wing morphology (high and low dispersal ability). We are not aware of other studies that have documented differences in carabid size distributions or wing morphology specifically as a result of changes in floral abundance. Gardens with more flowers (a more ephemeral resource than leaf litter) may attract beetles that can disperse across large distances. Carabid researchers have often predicted a higher abundance of smaller carabids in highly disturbed sites (i.e., the stress hypothesis [41,88]). Some studies found that the smaller carabid species were dominant in more disturbed urban environments, while larger species were dominant in more rural environments [23,41]. However, at least one study found that forest disturbance (i.e., flooding) fostered species diversity (contrary to the stress hypothesis) [89]. In our study system, greater amounts of urban cover in the landscape promoted abundance of larger carabids; therefore, we do not have evidence to support the stress hypothesis. Our results suggest that gardens surrounded by urban cover—often considered to be inhospitable habitat—may have local features that can support large beetles with brachypterous wings that cannot disperse across long distances. 

### 4.3. Implications for Pest Control in Urban Gardens

Our study results on the local and landscape drivers of carabid activity, richness and trait distribution in gardens can contribute useful information to gardeners who often lack knowledge regarding pest management in urban agroecosystems [90]. Most carabid beetles are predatory, but carabids can feed on a wide range of prey and plant material, depending on the life stage [26]. Predators with broad host ranges, like carabid beetles, are important contributors to biological control and lower pest abundances [91,92,93,94,95]. Two species that are common in our sites, *Pterostichus lustrans* and *T. obtusus*, are predators of common crop pests [96]. We found that gardens with greater crop species richness support a higher activity of carabids, and that large brachypterous beetles (i.e., those that provide greater pest control but are limited in dispersal ability) are affected by landscape surroundings. Further, a larger carabid body size boosts prey attack [97] and prey consumption rates [43]. Although carabid traits influence the dispersal ability and associated pest control, wing morphology and size alone do not determine carabid dispersal. Reproductive traits are also important; carabid females often lose functional flight musculature as their ovaries develop [98]. While large size is tied to higher prey consumption, small body size indicates a high reproductive rate, which is of high ecological importance for carabids in urban gardens [99]. Although pest control by carabids has been directly measured in rural systems (e.g., [43]), these nuances of carabid ecology call for future research that measures pest predation by carabids in urban agroecosystems.

## 5. Conclusions

Research has shown how local habitat factors influence carabid communities or how landscape impacts carabids in urban areas. However, most studies use a landscape approach (i.e., examining carabids along an urban to rural gradient), examine single habitat drivers (i.e., change in mulch), or examine differences in abundance, richness, or traits in non-garden urban habitats. Thus, our study contributes new information on carabid ecology by using an approach that examines multiple local and landscape environmental drivers in a single urban habitat type. We examine carabid activity-density and composition, and we also ask how functional traits relevant to pest control functions respond to these local and landscape drivers. Overall, we found relatively few carabids, which was either due to low activity or low sampling effort. Nonetheless, we can make some limited recommendations in how garden management and landscape surroundings might affect carabid composition and traits. We did not document strong effects of urban garden management or landscape surroundings on carabid taxonomic or functional richness, suggesting that regional scale effects of urbanization may be more important to the regional species pool. However, there were strong impacts of three local management factors (leaf litter, crop species, and floral abundance) and two landscape factors (agriculture cover and urban land cover) on activity density, community composition, and trait distributions in gardens. Thus, these factors, especially local factors that can be more easily manipulated by gardeners, could be used to boost the population of carabids, promote conservation goals, and encourage carabids that provide pest control in urban agroecosystems. 

## Figures and Tables

**Table 1:** Identity and functional traits of Carabidae beetle individuals collected from 19 urban garden sites in the central coast of California.

Species	No. Indiv.	No. Sites	Length (mm)	Size ^§^	Wing Morph-Ology ^§§^	Dispersal Ability ^¶¶^
*Amara (Amara) aenea* (DeGeer)	2	1	9	L	M	M
*Amara (Amara) littoralis* (Dejean)	5	4	8.8	L	M	H
*Amara (Celia) californica californica* (Dejean)	1	1	11	L	M	M
*Amara (Zezea) scitula* (Zimmermann)	1	1	11	L	M	M
*Anisodactylus californicus* (Dejan)	1	1	12	L	M	M
*Axinopalpus biplagiatus* (Dejean)	3	2	3.3	S	M	H
*Bembidion (Neja) ambiguum* (Dejean)	2	1	3.5	S	M	H
*Bradycellus (Liocellus) nitidus* (Dejean)	3	2	5	S	M	H
*Bradycellus (Stenocellus) nubifer* (LeConte)	4	3	4.5	S	M	H
*Calathus ruficollis ruficollis* (Dejean)	5	2	10.2	L	B	L
*Chlaenius (Chlaeniellus) tricolor vigilans* (Say)	3	1	15	L	M	M
*Harpalus (Pseudoophonus) pensylvanicus* (DeGeer)	8	2	17.5	L	M	M
*Laemostenus complanatus* (Dejean)	32	2	16.2	L	M	M
*Microlestes nigrinus* (Mannerheim)	3	2	3.7	S	D	M
*Notiobia (Anisotarsus) terminata* (Say)	3	2	12	L	M	M
*Poecilus (Poecilus) cursitor* (LeConte)	1	1	11	L	M	M
*Pterostichus (Bothriopterus) lustrans* (LeConte)	4	2	12.3	L	M	M
*Pterostichus (Hypherpes) californicus* (Dejean)	14	5	16.6	L	B	L
*Pterostichus (Hypherpes) vicinus* (Mannerheim)	3	2	16	L	M	M
*Trechus (Trechus) obtusus* (Erichson)	51	7	4.1	S	D	M

KWW identified all beetles at the Essig Entomology Museum at U. of California, Berkeley, CA. **^§^** Beetles were classified as small (S, <8 mm) and large (L, >8 mm). **^§§^** Macropterous (M), Dimorphic (D), Brachypterous (B). High (H), Medium (M), Low (L) dispersal ability (see text for calculation).

## Supplementary Materials

The following are available online at https://www.mdpi.com/2075-4450/10/4/112/s1, Table S1: Results from the two-step RLQ analysis. Eigenvalues and percentage of total co-inertia (%) for (a) preliminary ordinations and (b) the RLQ analysis. Table S2: Fourth corner correlations between local and landscape factors and carabid traits. Significant relationships (*p* < 0.05) are shown in bold.
