# Supplementary material for: Local and Landscape Drivers of Carabid Activity, Species Richness, and Traits in Urban Gardens in Coastal California
Source: Insects. 2019 Apr 19;10(4):112. doi: 10.3390/insects10040112 (PMC6523476; doi:10.3390/insects10040112)
Supplement: Supplementary file 1 [file insects-10-00112-s001.pdf]

*insects*

MDPI

*Article*

# Local and Landscape Drivers of Carabid Activity, Species Richness, and Traits in Urban Gardens in Coastal California

**Stacy M. Philpott [1,*], Simone Albuquerque [2], Peter Bichier [1], Hamutahl Cohen [3], Monika H. Egerer [1], Claire Kirk [1], and Kipling W. Will [4]**

[1] Environmental Studies Department, University of California, Santa Cruz, CA, 95062, USA; pbichier@ucsc.edu (P.B.); megerer@ucsc.edu (M.H.E.); clairekirk95@gmail.com (C.K.)
[2] Ecology and Evolutionary Biology Department, University of California, Santa Cruz, CA, 95062, USA; lostinalbuquerque@gmail.com
[3] Entomology Department, University of California, Riverside, CA, 92521, USA; hamutahc@ucr.edu
[4] Essig Museum of Entomology, University of California, Berkeley, CA 94720, USA; kipwill@berkeley.edu
* Correspondence: sphilpot@ucsc.edu; Tel.: +01-831-459-1549

Received: 22 March 2019; Accepted: 17 April 2019; Published: date

**Supplementary Material:**

**Table S1.** Results from the two-step RLQ analysis. Eigenvalues and percentage of total co-inertia (%) for (a) preliminary ordinations and (b) the RLQ analysis.

| | **Axis 1 (%)** | **Axis 2 (%)** |
|---|---|---|
| **(a) Preliminary ordinations** | | |
| R (PCA) | 2.60 (28.9%) | 2.06 (22.93%) |
| L (CA) | 0.92 (15.46%) | 0.81(13.73%) |
| Q (PCA) | 2.76 (46.00%) | 2.00 (33.35%) |
| **(b) RLQ analysis** | | |
| RLQ eigenvalues | 2.02 (74.62%) | 0.44 (16.31%) |
| Covariance | 1.42 | 0.66 |
| Correlations | 0.65 | 0.39 |
| Projected inertia R | 1.75 | 3.6 |
| Projected inertia Q | 2.76 | 4.3 |

**Table S2.** Fourth corner correlations between local and landscape factors and carabid traits. Significant relationships ($p < 0.05$) are shown in bold.

| Factor | Body length | Size class | Wing morph | Dispersal |
|---|---|---|---|---|
| Garden size | 0.88 | 0.93 | 0.95 | 0.11 |
| Bare soil | 0.89 | 0.96 | 0.39 | 0.75 |
| Mulch | 0.40 | 0.22 | 0.83 | 0.17 |
| Leaf litter | **0.005** | **0.04** | 0.17 | 0.69 |
| No. Flowers | **0.05** | **0.04** | **0.01** | 0.60 |
| No. crop species | 0.99 | 0.43 | 0.11 | 0.46 |
| No. weed species | 0.44 | 0.48 | 0.21 | 0.66 |
| Urban cover (2 km) | **0.03** | 0.06 | 0.12 | 0.68 |
| Agriculture cover (2 km) | 0.47 | 0.29 | 0.61 | 0.70 |

© 2019 by the authors. Licensee MDPI, Basel, Switzerland. This article is an open access article distributed under the terms and conditions of the Creative Commons Attribution (CC BY) license (http://creativecommons.org/licenses/by/4.0/).
